# Aberrant Volume-Wise and Voxel-Wise Concordance Among Dynamic Intrinsic Brain Activity Indices in Parkinson’s Disease: A Resting-State fMRI Study

**DOI:** 10.3389/fnagi.2022.814893

**Published:** 2022-03-29

**Authors:** Yuan Tian, Hai-Bo Chen, Xin-Xin Ma, Shu-Hua Li, Chun-Mei Li, Shao-Hui Wu, Feng-Zhi Liu, Yu Du, Kai Li, Wen Su

**Affiliations:** ^1^Department of Neurology, Beijing Hospital, National Center of Gerontology, Institute of Geriatric Medicine, Chinese Academy of Medical Sciences, Beijing, China; ^2^Graduate School of Peking Union Medical College, Dongcheng, Beijing, China; ^3^Department of Radiology, Beijing Hospital, National Center of Gerontology, Institute of Geriatric Medicine, Chinese Academy of Medical Sciences, Beijing, China

**Keywords:** Parkinson’s disease, resting-state fMRI, intrinsic brain activity, volume-wise concordance, voxel-wise concordance

## Abstract

Researches using resting-state functional magnetic resonance imaging (rs-fMRI) have applied different regional measurements to study the intrinsic brain activity (IBA) of patients with Parkinson’s disease (PD). Most previous studies have only examined the static characteristics of IBA in patients with PD, neglecting the dynamic features. We sought to explore the concordance between the dynamics of different rs-fMRI regional indices. This study included 31 healthy controls (HCs) and 57 PD patients to calculate the volume-wise (across voxels) and voxel-wise (across periods) concordance using a sliding time window approach. This allowed us to compare the concordance of dynamic alterations in frequently used metrics such as degree centrality (DC), global signal connectivity (GSC), voxel-mirrored heterotopic connectivity (VMHC), the amplitude of low-frequency fluctuations (ALFF), and regional homogeneity (ReHo). We analyzed the changes of concordance indices in the PD patients and investigated the relationship between aberrant concordance values and clinical/neuropsychological assessments in the PD patients. We found that, compared with the HCs, the PD patients had lower volume concordance in the whole brain and lower voxel-wise concordance in the posterior cerebellar lobe, cerebellar tonsils, superior temporal gyrus, and supplementary motor region. We also found negative correlations between these concordance alterations and patients’ age. The exploratory results contribute to a better understanding of IBA alterations and pathophysiological mechanisms in PD.

## Introduction

Parkinson’s disease (PD) is characterized by the decrease of dopaminergic neurons in the nigrostriatal pathway, resulting in a neurodegenerative disorder with progressive movement disorders (Kalia and Lang, 2015). Moreover, non-motor symptoms such as emotional disorders and cognitive impairment are also common in PD patients (Rana et al., 2015; Raza et al., 2019). Many neuroimaging studies have demonstrated that several regions of the brain are also impacted in PD patients (Weingarten et al., 2015). Resting-state functional magnetic resonance imaging (rs-fMRI) has also been widely used to study the IBA in PD, including both brain functional connectivity (FC; Tessitore et al., 2019) and regional cerebral neuronal activity (Yue et al., 2020), which has produced inconsistent results. Some meta-analyses of neuroimaging data have attempted to identify abnormalities in the consistency of IBA patterns in PD, such as studying the amplitude of low-frequency fluctuations (ALFF; Pan et al., 2017; Wang et al., 2018) or regional homogeneity (ReHo; Pan et al., 2016), and functional connectivity network (Tahmasian et al., 2017). There are also relevant studies on the degree centrality (DC; Guo et al., 2020; Chen et al., 2021), and voxel-minored homotopic connectivity (VMHC; Luo et al., 2015). However, most current rs-fMRI research in PD patients has focused on static characteristics of human brain activity and neglected the dynamic properties of IBA in the temporal dimension (Hutchison et al., 2013). In recent years, the sliding window approach has demonstrated the altered dynamic functional connectivity (dFC) of IBA in individuals with common neuropsychiatric disorders such as Alzheimer’s disease (de Vos et al., 2018), major depressive disorder (Hosenfeld et al., 2015), and primary generalized epilepsy (Liu et al., 2017).

Many studies have assessed dFC in PD and demonstrated that dynamical changes in IBA were associated with motor (Kim et al., 2017) and cognitive impairments (Fiorenzato et al., 2019). However, it is not enough to focus only on the dynamic functional connectivity features, because much information is found in the fluctuations of regional neural activity (Yu et al., 2019). Different rs-fMRI indicators represent different IBA aspects. The degree of integration is represented by the concordance between the different dynamic regional indices. Yan et al. investigated the volume-wise and voxel-wise concordance between these regional measures and found they demonstrated stable inter-individual differences that were correlated with age (Yan et al., 2017). Fu et al. found consistency in the changes in dynamic ALFF and dynamic FC over time, and this consistency differed between patients with schizophrenia and HCs (Fu et al., 2018). Whether the consistency between regional indicators is abnormal in PD patients remains a direction to be explored. Previous studies on dynamic indicators in PD have been performed with the dynamical amplitude of low-frequency fluctuation (dALFF) analysis (Zhang et al., 2019). Though the PD patients exhibit abnormalities in dynamic and static brain activity, the concordance between different dynamic regional indices remains unclear. In this study, we compared the differences in volume-wise and voxel-wise concordance among IBA measures between PD patients and HCs to explore the pathophysiological mechanisms of PD.

## Materials and Methods

### Participants

We recruited 67 individuals with PD and 34 healthy controls. Both groups were similar in age and gender and had no history of neurological or mental disorders. In this study, all the PD patients were diagnosed based on the diagnostic criteria established by the United Kingdom Parkinson’s Disease Society [17]. The Helsinki Declaration was closely followed at every step of this study. Before participating in the trial, all participants were required to provide written informed consent. This research was conducted according to all relevant regulations and laws and the study was authorized by the hospital’s ethical review committee. All of the individuals were right-handed. We have ruled out conditions such as deep brain stimulation, head trauma, moderate to severe tremors in the head, alcohol/drug abuse, dementia, and other mental and neurological issues. When all PD medications were stopped for 12 h, all patients with Parkinson’s disease were administered an MRI and non-motor/motor function tests in an “off” state: several demographic and clinical questions were posed to each PD participant. These tests included the Mini-Mental State Examination (MMSE), the Hamilton Depression Rating Scale (HAMD), the Hamilton Anxiety Rating Scale (HAMA), the Non-Motor Symptoms Questionnaire (NMSQ), the Unified Parkinson’s Disease Rating Scale (UPDRS), and the Hoehn-Yahr staging. Additionally, the MMSE was used to examine the participants in the control group.

### MRI Data Acquisition

MRI scans were performed on all patients at Beijing Hospital using a 3.0-T scanner (Achieva TX; Philips Medical Systems, Best, Netherlands). During the scan, the participants were instructed to remain motionless, relax, close their eyes, and maintain their alertness. The use of foam cushion and headphones helped to keep the scanning noise and head movements to a minimum. The following settings were used to generate high-resolution T1-weighted images (three-dimensional turbo field echo): field of view (FOV) = 240 × 240 mm, echo time (TE) = 3.0 ms, repetition time (TR) = 7.4 ms, flip angle = 8°, slice thickness = 1.2 mm, matrix size = 256 × 256, voxel dimensions = 0.94 × 0.94 × 1.20 mm, 140 slices. The functional pictures were generated using the following parameters: FOV = 240 × 240 mm, flip angle = 90, TR = 3,000 ms, TE = 35 ms, voxel dimensions 3.75 × 3.75 × 4.00 mm, matrix size = 64 × 64, slices = 33, time points = 210, slice thickness = 4 mm.

### Resting-State Functional Magnetic Resonance Imaging Data Preprocessing

Data preprocessing for the rs-fMRI was performed using RESTPlus V 1.2 (Jia et al., 2019) and the SPM12 program^1^ in MATLAB (MathWorks, Inc., Natick, MA, United States). Ten volumes were initially eliminated to stabilize the magnetization and adjust it to the subject’s magnetic fields. Participants with head motion above 2 mm in displacement or 2° in rotation were then excluded from the study (Supplementary Table 1). The remaining 200 volumes were adjusted according to the slice time and readjusted to account for the head motion. Individual T1WI pictures were co-registered to the mean functional images, and then segmented into the cerebrospinal fluid (CSF), white matter, and gray matter. The functional volume of each individual was segmented and then normalized to the Montreal Neurological Institute (MNI) space using the Diffeomorphic Anatomical Registration Through Exponentiated Lie Algebra (DARTEL) toolbox for two-dimensional anatomical registration. They were then resampled to 3 mm isotropic voxels (Ashburner, 2007), which were used to determine the functional volumes of each individual. The rs-fMRI indices were extracted after functional volumes were smoothed with a 6-mm full width at half maximum (FWHM) Gaussian kernel, and the linear trend of the time course was eliminated. The volumes were then filtered with a bandpass filter between 0.01 and 0.08 Hz. The nuisance covariate regression included the Friston 24-head motion parameters, white matter and CSF signals (Power et al., 2012).

### Calculation of Dynamic Resting-State Functional Magnetic Resonance Imaging Measures

The dynamic rs-fMRI measures and their concordance were computed using DPABI V6.0 (Yan et al., 2016). Sliding time window analysis was used to examine the temporal dynamics of the five rs-fMRI measurements listed below. The preprocessed functional data from each participant was subjected to hamming windows (window size = 30 TR; window step = 1 TR) in order to produce a series of BOLD signal windows. For each window, the dynamics of these rs-fMRI measurements were then calculated (Supplementary Figures 1, 2).

(1) Amplitude of low-frequency fluctuation (ALFF): We used a Fast Fourier Transform to create the power spectrum by transforming the BOLD time course to a frequency domain (FFT). In this study, ALFF was defined as the mean power spectrum in a particular low-frequency band (0.01–0.1 Hz) (Zang et al., 2007), while fALFF was defined as the ratio of the power spectrum in the low-frequency band (0.01–0.1 Hz) to the entire power spectrum in the frequency range (Zou et al., 2008). Because ALFF is sensitive when evaluating aberrant brain activity in individuals with neuropsychiatric illnesses, we only used ALFF in the concordance analysis (de Vos et al., 2018).

(2) Regional homogeneity (ReHo): Regional brain activity was quantified using the ReHo method, which presumes that neural activity is more likely to occur in a cluster than in a single voxel, and is thus more consistent. Kendall’s coordination coefficient (Kendall’s W) was obtained when the time series of a particular voxel was compared with the time series of its nearest neighbors (Zang et al., 2004).

(3) Voxel-mirrored homotopic connectivity (VMHC): To enhance the relationship between symmetric voxels, we converted the functional pictures to a symmetric space from a functional image space. We then averaged all of the normalized T1 pictures to obtain a mean T1 image. This was then averaged with its left and right mirror counterparts to create a group-specific symmetrical template using the mean T1 image as a guide. The non-linear registration of each subject was then adjusted to the standard space using the symmetric template and the functional data of each subject was changed to the symmetric space using the symmetric template. The Pearson’s correlation coefficient between the time series of the symmetric inter-hemispheric counterpart and the time series of each voxel was defined as the Pearson’s correlation coefficient between any pair of symmetric inter-hemispheric voxels (Zuo et al., 2010).

(4) Degree centrality (DC): DC is defined as the number of edges connecting a node (binary graphs) or the total of their weights (weighted graphs). The Pearson correlation coefficients were between the BOLD time courses of all pairs of voxels, after which a gray matter functional connectivity matrix was constructed for each participant. The DC was calculated as the total number of positive functional connections larger than the threshold of 0.25 between a specific voxel and all other voxels in the gray matter surrounding that voxel (Zuo et al., 2012).

(5) Global signal connectivity (GSC): The gray matter correlation coefficient (GSC) was determined as the Pearson’s correlation coefficient between the mean time course and the BOLD time course of each voxel throughout the whole gray matter. The Fisher Z transformation was then applied to the resulting GSC maps for further analysis (Hahamy et al., 2014).

### Volume-Wise and Voxel-Wise Concordance

Since Kendall’s W is a non-parametric statistic that does not require distributional assumptions and is not impacted by scale discrepancies between these rs-fMRI indices, it was used to quantify the volume and voxel consistency among them (Yan et al., 2017). We calculated two kinds of consistency among the five abovementioned dynamic indices: (1) the volume-wise consistency index is an index reflecting global-level consistency and used Kendall’s W to calculate rs-fMRI indices in each subject’s brain in the scan and across all time windows; (2) the voxel-wise concordance index used Kendall’s W to calculate each voxel coordination across time windows and provided an interactive voxel concordance map (Yan et al., 2017).

### Statistical Analysis

A two-sample *t*-test was used to assess the demographic information and neuropsychological scale scores of each group. The quantitative data were showed as a mean ± standard deviation. The chi-square test was used to compare participant gender characteristics across groups, while the inter-group volume-wise concordance was also analyzed using a two-sample *t*-test. Clinical data were analyzed using SPSS (SPSS Version 21.0. Inc., Chicago, IL, United States). *P* < 0.05 was considered significant. The normalized voxel-wise concordance maps of each rs-fMRI index were then compared between the two groups using ANCOVA analysis using DPABI V6.0. Age and gray matter volume (GMV) were included as covariates and subsequently adjusted by regression throughout the statistical analysis to reduce the impact of confounding factors. The voxel-level and cluster-level *P* < 0.05 significance levels were used to correct multiple comparisons using a Gaussian random field correction (GRF) with a voxel-level *P* < 0.001 and cluster-level *P* < 0.05 (Chen et al., 2018). The concordance indices were extracted from areas with significant between group differences and the correlations between the extracted concordance indices values and HAMA, HAMD, NMSQ, MMSE, UPSR scores, H-Y staging, and disease duration were assessed using Spearman correlation analyses.

## Results

### Demographic and Clinical Characteristics

This research assessed a total of 57 individuals with PD and 31 HCs. Four PD patients and two controls were excluded from the study because they were left-handed. Five PD patients and one healthy control were omitted from the study due to excessive head motion. One PD disease patient was excluded from the study due to low picture quality. Table 1 summarizes the demographic and clinical data. No statistically significant differences were found in the MMSE scores, age, and gender between the PD and the HCs (*p* > 0.05). Table 1 summarizes demographic and clinical data. There were no differences in the age, gender, or MMSE scores between PD patients and HCs (*p* > 0.05).

**TABLE 1:** Clinical and demographic characteristics of the Parkinson’s disease (PD) patients and the healthy controls (HCs).

	PD	HCs	*p*-value
No. of subjects	57	31	
Age	64.16 ± 8.13	62.42 ± 7.19	0.322
Gender (male/female)	28/29	16/15	0.823
Disease duration	6.49 ± 3.59	N/A	N/A
HAMA	10.21 ± 5.49	N/A	N/A
HAMD	9.30 ± 5.14	N/A	N/A
MMSE	28.05 ± 1.95	27.71 ± 2.25	0.458
NMSQ	11.30 ± 5.32	N/A	N/A
H-Y staging	2.20 ± 0.69	N/A	N/A
UPDRS	49.90 ± 18.82	N/A	N/A

*PD, Parkinson’s disease; HCs, healthy controls; M, male; F, female; MMSE, mini-mental status examination; HAMA, hamilton anxiety rating scale; HAMD, hamilton depression rating scale; NMSQ, non-motor symptoms questionnaire; H-Y staging, Hoehn-Yahr staging; UPDRS, unified Parkinson’s disease rating scale.*

### Volume-Wise Concordance Alterations in Parkinson’s Disease

**FIGURE 1:**
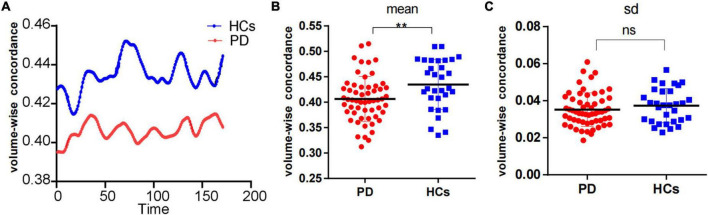
Comparison of volume-wise concordance indices between PD patients and HCs. **(A)** Volume-wise concordance time series of the mean value in PD patients and HCs. **(B)** Comparison of mean volume-wise concordance indices. **(C)** Comparison of the SD of volume-wise concordance indices. PD, Parkinson’s disease; HCs, healthy controls. SD, Standard deviation. **P < 0.01; ns, no significance.

**FIGURE 2:**
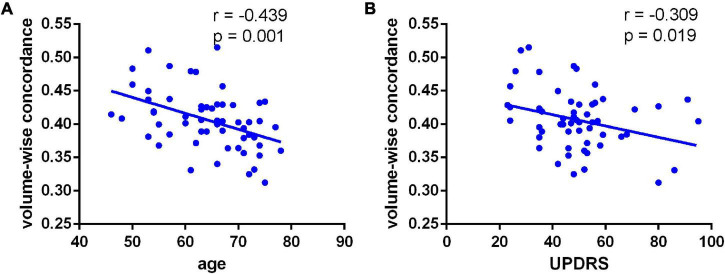
Negative associations between volume-wise concordance and age **(A)** UPDRS **(B)** scores in individuals with PD (*r* = –0.439, –0.309; *P* = 0.001, 0.019).

We observed a significant degree of volume-wise concordance across the whole brain in the HCs and PD patients (mean Kendall’s *W* ≥ =0.4), indicating a high degree of spatial distribution consistency across these rs-fMRI measurements. The mean value of volume consistency significantly differed between the two groups (*P* = 0.007), indicating a statistically significant difference between the two groups. However, there was no statistically significant difference in the standard deviation (SD) of volume-wise concordance (*P* = 0.299) (Figure 1 and Table 2). We also identified statistically significant negative associations between volume-wise concordance and age/UPDRS scores in individuals with PD (*r* = =−0.439, −0.309; *P* = =0.001, 0.019) (Figure 2).

**TABLE 2:** Comparison of volume-wise concordance indices between PD patients and HCs.

	PD	HCs	P
Mean	0.41 ± 0.04	0.44 ± 0.05	0.007
SD	0.035 ± 0.01	0.037 ± 0.01	0.299

*PD, Parkinson’s disease; HCs, healthy controls; SD, standard deviation.*

### Voxel-Wise Concordance Alterations in Parkinson’s Disease

**FIGURE 3:**
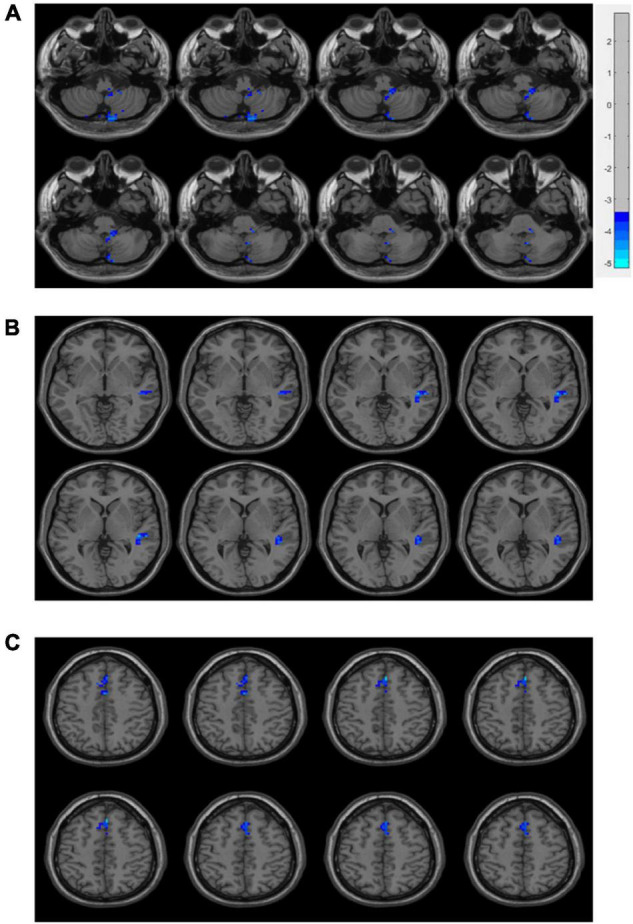
Brain regions of voxel-wise concordance differences between PD patients and HCs: the cerebellum posterior lobe (CPL) and cerebellar tonsil (CTO) **(A)**, superior temporal gyrus (STG) **(B)**, and supplementary motor area (SMA) **(C)**.

**FIGURE 4:**
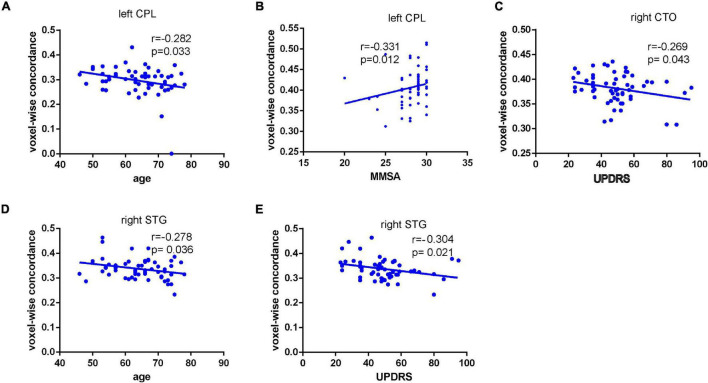
Connection between age/MMSA scores and voxel-wise concordance value in the left CPL in PD patients from HCs (*r* = –0.282,0.331; *P* = 0.033, 0.012). **(A,B)** Negative correlation between concordance value in the CTO and UPDRS scores (*r* = –0.269; *P* = 0.043). **(C)** Negative association between age, UPDRS scores, and the concordance value in STG (*r* = –0.278, –0.304; *P* = 0.036, 0.021) **(D,E)**.

As displayed in Figure 3, inter-group comparisons indicated that PD patients exhibited worse voxel-wise concordance than the HCs in the following regions: the cerebellum posterior lobe (CPL), cerebellar tonsil (CTO), superior temporal gyrus (STG), and supplementary motor area (SMA) (GRF, voxel level, *p* < 0.001; cluster level, *p* < 0.05) (Figure 3 and Table 3). Additionally, the Spearman correlation analyses demonstrated a connection between age/MMSA scores and voxel-wise concordance values in the left CPL between PD patients and HCs (*r* = =−0.282, 0.331; *P* = 0.033, 0.012) (Figure 4A). The concordance value in the CTO is negatively correlated with UPDRS scores (*r* =−0.269; *P* = 0.043) (Figure 4B), and there is a negative association between age, UPDRS scores, and the concordance values in STG (*r* = −0.278, −0.304; *P* = 0.036, 0.021) (Figures 4C–E).

**TABLE 3:** Brain regions of voxel-wise concordance differences between PD patients and HCs.

Brain regions	Size (voxels)	MNI coordinates (mm)			Peak value
		X	Y	Z	
Right CPL	142	18	−75	−54	−5.1893
Left CPL	69	−18	−69	−57	−4.5713
Right CTO	32	6	−57	−39	−4.7521
Right STG	51	45	−27	0	−4.8072
SMA	83	3	27	48	−4.7717

*CPL, cerebellum posterior lobe; CTO, cerebellar tonsil; STG, superior temporal gyrus; SMA, supplementary motor area.*

## Discussion

Concordance abnormalities in rs-fMRI brain activity data were significantly altered in PD patients based on a dynamic analytic technique. PD patients had worse voxel concordance in certain cerebral regions and lower volume concordances throughout the whole brain than did the healthy controls. Furthermore, age was related to changes in volume concordance, and motor symptoms and age were associated with decreases in voxel concordance regions. This demonstrates that coordination in Parkinson’s patients is clinically meaningful.

Previous research has applied various static and dynamic indicators to explore the characteristics of spontaneous brain activity in PD patients, which provided helpful information about certain brain regions (Li et al., 2018; Wang et al., 2018; Filippi et al., 2019). However, certain regions demonstrated various degrees of dysfunction, and some measures were the only way of detecting functional impairments in certain brain regions. The distinctions and overlaps between these rs-fMRI measures remain unknown. Resting-state indicators are constantly being developed, leading researchers to examine the consistency between multiple indices. Therefore, based on the dynamic nature of IBA, we further investigated the concordance of different rs- fMRI indices. Different indices reflect different aspects of IBA, which means that their concordance can be measured by reflecting the integration of different functional levels (Yan et al., 2017). The volume-wise concordance demonstrates the consistency of the spatial distribution of these rs-fMRI values. As previously reported (Yan et al., 2017), a high degree of spatial distribution consistency was observed with high volume-wise concordance in HCs (mean Kendall’s *W* > 0.4). We also observed volume-wise concordance in PD patients (mean Kendall’s W≈0.4), which was significantly lower than HCs. This indicates a less consistent spatial distribution, which could be due to the inconsistent pattern of spatial variation of rs-fMRI measurements in PD. Moreover, the inter-voxel agreement reflects the concordance of temporal dynamics between these rs-fMRI measures. In this study, the frontal lobe, temporal gyrus, and cerebellum of PD patients displayed decreased voxel-wise concordance. The reason for voxel-wise concordance disruptions could be the inconsistent pattern of temporal dynamic changes in rs-fMRI measurements. For detecting regional functional abnormalities, voxel-wise concordance may be the novel symbols based on the single rs-fMRI measurements. For example, concordance studies have been performed on schizophrenia (Zhu et al., 2018), subjective cognitive decline (SCD; Yang et al., 2021), and Alzheimer’s disease (AD; Chen et al., 2021), which have identified abnormalities in spontaneous dynamic brain activity and integration.

The SMA is located on the medial side of the cerebral hemisphere, in front of the primary motor cortex. It is closely related to motor planning, learning, and cognitive functions (Nachev et al., 2008). The SMA is connected to the basal ganglia through the thalamus, forming the striatal-thalamocortical loop (STC loop). On the other hand, the SMA connects to the cerebellum through the thalamus to form the cerebellum-thalamocortical loop (CTC loop). Studies have demonstrated that the primary pathogenesis of PD is damage to the STC loop, which disrupts the intracranial control circuit and causes a relatively active CTC loop to compensate for functional deficits caused by this damage (Palmer et al., 2010). PD patients have impaired SMA function, which decreases connectivity between the SMA and other functional brain areas and results in motor impairments in PD patients (Yu et al., 2007; Prodoehl et al., 2010; Wu et al., 2010).

Parkinson’s disease patients struggle in rhythm recognition assignments. The right STG plays a crucial role in this process and increased activity in the right STG region suggests inhibited temporal auditory perception (Vikene et al., 2019). The medial temporal lobe is an important center of emotional integration and is associated with motor control, self-perception, and emotional regulation (Paus et al., 1996). While PD has typically been characterized by motor impairment, it can also produce several different non-motor symptoms (Reichmann et al., 2016). We found a significant correlation between the UPDRS score and the voxel-wise concordance value of STG. This could represent enhanced temporal lobe activity as a compensatory mechanism for impaired motor symptoms.

The cerebellum coordinates somatic balance, muscle tone, and casual movements, and is a key node in the CTC loop, controlling and influencing somatic movements (Yang et al., 2013; Hanakawa et al., 2017). Prior studies have found that neuronal activity in cerebellar hemispheres increases in patients with PD compared with HCs (Yu et al., 2007), and that deep stimulation of the subthalamic nuclei inhibits glucose metabolism in the cerebellum (Hilker et al., 2004). Therefore, increased neuronal activity in the cerebellum of PD patients could be functional compensation for the hypofunction of the basal ganglia (Lewis et al., 2007). Several studies have demonstrated that Parkinson’s tremor is closely related to the CTC loop (Hacker et al., 2012; Helmich et al., 2012; Zhang et al., 2015). Additionally, pathological and compensatory effects are believed to result from the cerebellum in PD patients. Certain clinical symptoms could be caused by pathological cerebellar alterations resulting from degeneration of aberrant drives from the basal ganglia, dopaminergic system, and dopaminergic therapy. This compensatory effect could help maintain both functional motor and non-motor systems (Wu and Hallett, 2013). Additionally, recruitment of the CTC circuit is positively related with the severity of symptoms or development in PD of mild to moderate stages (Sen et al., 2010). Neuronal activity in the cerebellar hemispheres is increased in patients with PD compared to HCs (Yu et al., 2007), and its deep stimulation of the subthalamic nuclei clusters aligns glucose metabolism in the cerebellar brain (Hilker et al., 2004). Pathological damages increase in severity as the disease progresses, while the compensatory effect may weaken or fail altogether (Jankovic, 2005). This study demonstrates that concordance indexes in the posterior cerebellar lobes and cerebellar tonsil decreased in PD patients and that concordance values are correlated with age, motor, and non-motor symptoms. This indicates that neuronal activity increased, which compensates for decreases in the STC loop function.

## Limitation

This study has some limitations. First, the size of this study sample was insufficient; therefore, a study with a larger number of participants is necessary. For the concordance studies, we only used five common rs-fMRI measures since these data-driven measures are less likely to be influenced by variables such as component selection in independent component analysis and seed specification in seed-based correlation analysis. Accounting for additional rs-fMRI measurements could allow for better IBA characteristics and a more nuanced understanding of the physiological mechanisms responsible for these activities.

## Conclusion

In conclusion, this study demonstrates that PD patients have altered patterns of coherence obtained from rs-fMRI across several routine IBA measurements. We believe that concordance measures can provide novel insights into IBA mechanisms in PD patients.

## Data Availability Statement

The raw data supporting the conclusions of this article will be made available by the authors, without undue reservation.

## Ethics Statement

The studies involving human participants were reviewed and approved by Beijing Hospital. The patients/participants provided their written informed consent to participate in this study.

## Author Contributions

WS, H-BC, and KL conceived and designed the experiments. YT and KL analyzed the fMRI data. C-ML was responsible for the fMRI scan and helped perform fMRI data analyses. WS, S-HL, and H-BC recruited the subjects. X-XM, F-ZL, S-HW, and YD collected the demographic, clinical, and neuropsychological information of the subjects. YT, KL, and WS wrote the manuscript. All authors read and approved the final manuscript.

## Conflict of Interest

The authors declare that the research was conducted in the absence of any commercial or financial relationships that could be construed as a potential conflict of interest.

## Publisher’s Note

All claims expressed in this article are solely those of the authors and do not necessarily represent those of their affiliated organizations, or those of the publisher, the editors and the reviewers. Any product that may be evaluated in this article, or claim that may be made by its manufacturer, is not guaranteed or endorsed by the publisher.

## Footnotes

1
www.fifil.ion.ucl.ac.uk/spm
