# Supplementary figures and images for: Aberrant Volume-Wise and Voxel-Wise Concordance Among Dynamic Intrinsic Brain Activity Indices in Parkinson’s Disease: A Resting-State fMRI Study
Source: Front Aging Neurosci. 2022 Mar 29;14:814893. doi: 10.3389/fnagi.2022.814893 (PMC9004459; doi:10.3389/fnagi.2022.814893)

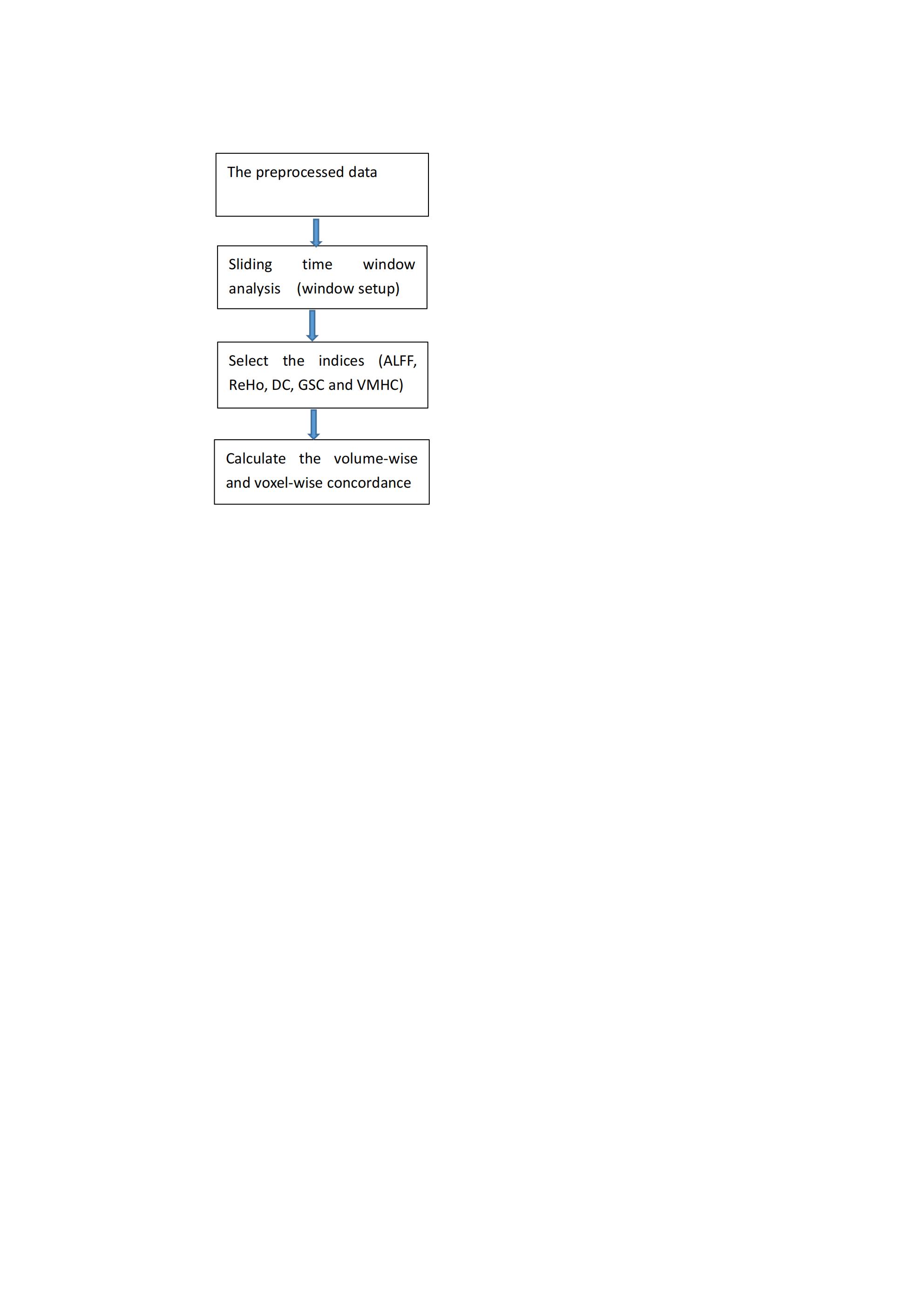

Supplement: Supplementary file 1 [file Image_1.JPEG]

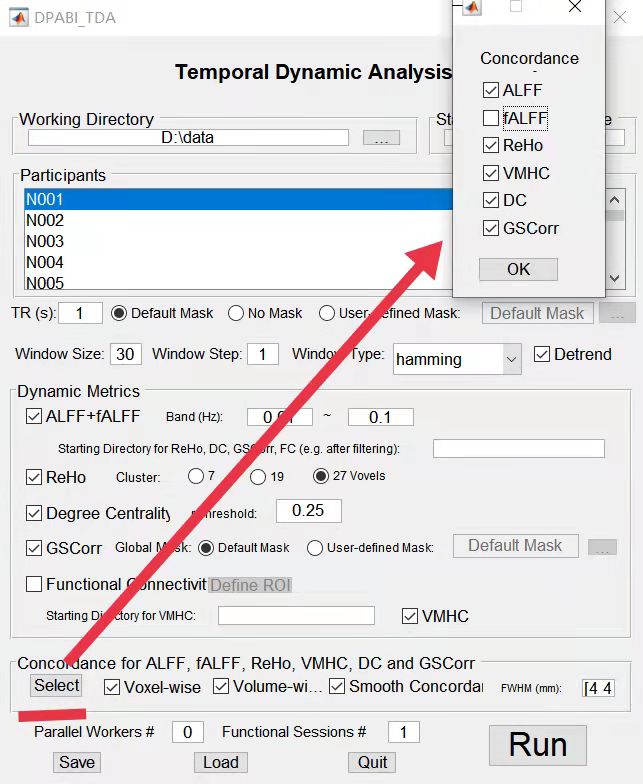

Supplement: Supplementary file 2 [file Image_2.JPEG]
