# Supplementary material for: Aberrant Volume-Wise and Voxel-Wise Concordance Among Dynamic Intrinsic Brain Activity Indices in Parkinson’s Disease: A Resting-State fMRI Study
Source: Front Aging Neurosci. 2022 Mar 29;14:814893. doi: 10.3389/fnagi.2022.814893 (PMC9004459; doi:10.3389/fnagi.2022.814893)
Supplement: Supplementary file 3 [file Table_1.DOCX]

Supplementary material

Table 1 headmotion parameters

|  | Right | Forward | Up | Pitch | Roll | Yaw |
| --- | --- | --- | --- | --- | --- | --- |
| 1 | 0.1280098 | 0.070450533 | 0.086948549 | 0.12870265 | 0.137983002 | 0.140496293 |
| 2 | 0.075937455 | 0.1023019 | 0.39703069 | 0.169846457 | 0.130875438 | 0.08926769 |
| 3 | 0.23434987 | 0.37011906 | 0.51965875 | 0.439284559 | 0.248008254 | 0.411907037 |
| 4 | 0.75750647 | 0.23435291 | 0.29175613 | 0.633099399 | 0.359032887 | 0.412655829 |
| 5 | 0.36971213 | 0.48555441 | 2.3712936 | 1.861305136 | 0.743730215 | 0.474268107 |
| 6 | 0.066121269 | 0.19344927 | 0.1703134 | 0.3565287 | 0.131990047 | 0.117948588 |
| 7 | 0.12241308 | 0.1363549 | 0.23926782 | 0.305889762 | 0.103572022 | 0.087887864 |
| 8 | 0.3818503 | 0.10064685 | 0.1905397 | 0.270462831 | 0.216850442 | 0.229590749 |
| 9 | 0.20810229 | 0.12561929 | 0.26971358 | 0.191936066 | 0.186560427 | 0.073033194 |
| 10 | 0.29124061 | 0.20593835 | 0.24188102 | 0.353419041 | 0.373924851 | 0.206858711 |
| 11 | 0.17073158 | 0.58099933 | 0.36256458 | 1.42592694 | 0.382397229 | 0.403225993 |
| 12 | 0.12533138 | 0.87420422 | 0.17017172 | 0.73982356 | 0.116016327 | 0.124938604 |
| 13 | 0.13302368 | 0.33359454 | 0.22844999 | 0.398623417 | 0.111208851 | 0.106790773 |
| 14 | 0.098262923 | 0.15057086 | 0.13006762 | 0.286273922 | 0.06154755 | 0.0735834 |
| 15 | 0.29633863 | 0.41004241 | 0.44055042 | 0.632521399 | 0.22716637 | 0.438728314 |
| 16 | 0.17146463 | 0.17343531 | 0.33912752 | 0.249604617 | 0.111150208 | 0.138440727 |
| 17 | 0.18640909 | 0.40693619 | 0.19661105 | 0.505023765 | 0.198268172 | 0.146358144 |
| 18 | 0.091907481 | 0.17486845 | 0.084995637 | 0.10832143 | 0.130405183 | 0.115442075 |
| 19 | 0.16492246 | 0.22715238 | 0.53629208 | 0.312841688 | 0.17011938 | 0.110435197 |
| 20 | 0.1220656 | 0.18455102 | 0.23325167 | 0.381135971 | 0.180852283 | 0.115659713 |
| 21 | 0.082325694 | 0.073470807 | 0.19037613 | 0.094558892 | 0.115708271 | 0.153070356 |
| 22 | 0.10172866 | 0.35671971 | 0.17221975 | 0.525952263 | 0.050012362 | 0.298898056 |
| 23 | 0.13169474 | 0.1620909 | 0.2491083 | 0.414294208 | 0.232255627 | 0.104234774 |
| 24 | 0.23850339 | 0.12815453 | 0.1621662 | 0.220489297 | 0.139367429 | 0.087991948 |
| 25 | 0.30510069 | 0.4683215 | 1.0150557 | 1.390431517 | 0.489473496 | 0.487791956 |
| 26 | 0.39747948 | 0.65078098 | 0.55458154 | 0.834480026 | 0.528632995 | 0.456184006 |
| 27 | 0.067450381 | 0.097520876 | 0.08083258 | 0.103647607 | 0.048725472 | 0.078953109 |
| 28 | 0.40909406 | 0.39260733 | 0.706726 | 0.370791282 | 0.420353638 | 0.171892776 |
| 29 | 0.20122128 | 0.25389396 | 2.388103 | 1.074955512 | 0.356865491 | 0.263477438 |
| 30 | 0.22498219 | 0.31420859 | 0.34619261 | 0.387623298 | 0.177820993 | 0.34662929 |
| 31 | 0.41794079 | 0.52944638 | 0.56027214 | 0.963845302 | 0.551712062 | 0.60950196 |
| 32 | 0.043406915 | 0.12412721 | 0.072610357 | 0.161281764 | 0.047233134 | 0.069422431 |
| 33 | 0.12015706 | 0.1187569 | 0.16922339 | 0.185223372 | 0.12310177 | 0.116827756 |
| 34 | 1.6701044 | 0.48922653 | 0.39052117 | 0.529785076 | 1.342726822 | 0.689556368 |
| 35 | 0.17610042 | 0.19397804 | 0.16815253 | 0.490805508 | 0.159296665 | 0.219720159 |
| 36 | 0.14550685 | 0.24285472 | 0.85494477 | 0.263117283 | 0.107770726 | 0.199594913 |
| 37 | 0.080911344 | 0.24205848 | 0.74295263 | 0.587350839 | 0.184201995 | 0.078538826 |
| 38 | 0.16852648 | 0.33448823 | 0.2392256 | 0.680508562 | 0.138711701 | 0.291963479 |
| 39 | 0.24313868 | 0.5363348 | 0.55181271 | 1.066055816 | 0.258274002 | 0.25376385 |
| 40 | 0.088548339 | 0.18119832 | 0.1999373 | 0.166561014 | 0.129002508 | 0.137299618 |
| 41 | 0.25496089 | 0.26035154 | 0.1723096 | 0.238880291 | 0.183690384 | 0.122069758 |
| 42 | 0.097228389 | 0.12043621 | 0.22193888 | 0.148328259 | 0.109131145 | 0.101891228 |
| 43 | 0.61326471 | 0.68020648 | 0.69558868 | 0.737145498 | 0.671431135 | 0.436423883 |
| 44 | 0.053462467 | 0.073560626 | 0.091048555 | 0.123517766 | 0.043795699 | 0.040490846 |
| 45 | 0.97677379 | 0.92176639 | 1.041793 | 2.292801943 | 1.273626565 | 2.038760077 |
| 46 | 0.38432054 | 0.52805153 | 0.84720364 | 1.097357131 | 0.331817564 | 0.492018253 |
| 47 | 0.47700859 | 0.42055752 | 0.81041274 | 0.656399873 | 0.665413474 | 0.744931421 |
| 48 | 0.22512815 | 0.12613598 | 0.14747889 | 0.143031648 | 0.19524182 | 0.083060889 |
| 49 | 0.088237269 | 0.13128516 | 0.078795016 | 0.199743962 | 0.10215052 | 0.068080684 |
| 50 | 0.082582422 | 0.12924811 | 0.35074243 | 0.398511415 | 0.166318682 | 0.190057818 |
| 51 | 0.22118166 | 0.37236013 | 0.39153412 | 0.505405366 | 0.298465008 | 0.180149172 |
| 52 | 0.37501065 | 0.28372419 | 0.38871213 | 0.626804197 | 0.473078016 | 0.42484911 |
| 53 | 0.67334614 | 0.75443471 | 2.8391518 | 4.24293771 | 0.788245483 | 0.698051785 |
| 54 | 0.21753049 | 0.49749442 | 0.65134705 | 1.575981436 | 0.3701356 | 0.457359297 |
| 55 | 0.71719242 | 0.94529879 | 0.36218838 | 1.704296537 | 1.084163861 | 0.537744709 |
| 56 | 0.21540267 | 0.19574916 | 0.098728318 | 0.200280657 | 0.133072433 | 0.145040822 |
| 57 | 0.16032145 | 0.37347192 | 0.092831468 | 0.575652358 | 0.233392375 | 0.19748322 |
| 58 | 0.13570009 | 0.1541301 | 0.15928287 | 0.383944141 | 0.215769574 | 0.081737506 |
| 59 | 0.52093142 | 0.27990898 | 0.60152754 | 0.585547855 | 0.667192909 | 0.352234055 |
| 60 | 0.22472404 | 0.37002287 | 0.24869148 | 0.67331989 | 0.151069209 | 0.329497537 |
| 61 | 0.30149863 | 0.26485834 | 0.22308018 | 0.512083006 | 0.308771717 | 0.282018668 |
| 62 | 0.22169326 | 0.45062599 | 1.3136224 | 0.665224226 | 0.301625495 | 0.198371917 |
| 63 | 0.094100265 | 0.096280631 | 0.11943009 | 0.295401781 | 0.110109923 | 0.13536125 |
| 64 | 0.20444079 | 0.18727898 | 0.19968411 | 0.21224966 | 0.198280817 | 0.129189991 |
| 65 | 0.092998057 | 0.10757096 | 0.094839011 | 0.115696365 | 0.144298258 | 0.105861424 |
| 66 | 0.275177 | 0.13473053 | 0.142073 | 0.278330939 | 0.230590348 | 0.332215007 |
| 67 | 0.20134559 | 0.21339419 | 0.14334401 | 0.336666545 | 0.204209991 | 0.200699776 |
| 68 | 0.31958239 | 0.2587569 | 0.29025725 | 0.214990581 | 0.192604249 | 0.20540451 |
| 69 | 0.45519594 | 0.088960166 | 0.17939048 | 0.377684078 | 0.288576611 | 0.46924596 |
| 70 | 0.25119508 | 0.21778452 | 0.27289931 | 0.226720236 | 0.203795307 | 0.140467061 |
| 71 | 0.15099714 | 0.11810112 | 0.15412195 | 0.13778532 | 0.188945066 | 0.124340631 |
| 72 | 1.0577374 | 1.0795178 | 0.70819609 | 1.767634214 | 0.765314853 | 1.582965791 |
| 73 | 0.19862484 | 0.17914533 | 0.16161925 | 0.292704359 | 0.177105053 | 0.251020912 |
| 74 | 0.30087506 | 0.22787052 | 1.6469714 | 0.785741601 | 0.5673533 | 0.334943512 |
| 75 | 0.66124345 | 0.61672859 | 0.30804687 | 0.838841782 | 0.613110563 | 0.179551566 |
| 76 | 0.33414975 | 0.30800878 | 0.50179891 | 0.279222754 | 0.231215726 | 0.289627249 |
| 77 | 0.15817873 | 0.15171844 | 0.28025733 | 0.324668901 | 0.221056256 | 0.128418383 |
| 78 | 0.21129365 | 0.45095301 | 1.6643973 | 0.465766765 | 0.205813063 | 0.210666663 |
| 79 | 0.42003858 | 0.26969232 | 0.64271917 | 0.506547174 | 0.62884003 | 0.291348563 |
| 80 | 0.21987026 | 0.12727452 | 0.15583831 | 0.125470051 | 0.170632933 | 0.187191173 |
| 81 | 0.24821651 | 0.3361564 | 0.36924817 | 0.547721222 | 0.238454079 | 0.249581756 |
| 82 | 0.49209723 | 0.18177754 | 0.23397199 | 0.501334152 | 0.397428869 | 0.470570077 |
| 83 | 0.1390945 | 0.28360505 | 2.1156096 | 0.609345657 | 0.265535566 | 0.140230962 |
| 84 | 0.46924591 | 0.18029047 | 0.42475633 | 0.325175997 | 0.499172852 | 0.333657956 |
| 85 | 1.5861106 | 0.98589536 | 0.53432385 | 1.341128671 | 0.727141826 | 2.07496803 |
| 86 | 0.16243163 | 0.13867322 | 0.27991237 | 0.29017687 | 0.152446514 | 0.147634299 |
| 87 | 0.4095969 | 0.24694431 | 0.27162977 | 0.43599228 | 0.256183937 | 0.278883907 |
| 88 | 0.34583039 | 0.2181743 | 0.31560262 | 0.314264532 | 0.380672758 | 0.323894686 |

Figure 1. The flow chart of the concordance indicator calculation method.

1. Data preprocessing for the rs-fMRI was performed using RESTPlus V 1.2 and the SPM12 program in MATLAB.
2. Sliding time window analysis (window size = 30 TR; window step = 1 TR) was used to examine the temporal dynamics of the selected rs-fMRI measurements.
3. The preprocessed functional data was subjected to hamming windows in order to produce a series of BOLD signal windows. For each window, the dynamics of these rs-fMRI measurements were then calculated.
4. The volum-wise concordance and voxle-wise concordance among these five selected measurements were computed (Supplementary material-figure 1 and figure 2).

Figure 2. The dpabi data dynamic analysis processing window:

1. Select the preprocessed data folder as the starting directory for dynamic analysis.
2. Set up the hamming windows (window size = 30 TR; window step = 1 TR) and select five rs-fMRI measurements.
